# The human cost of care: the relationship between fatigue and work capacity in Brazilian and Portuguese nursing professionals

**DOI:** 10.3389/fpubh.2026.1829368

**Published:** 2026-06-16

**Authors:** Clenise Liliane Schmidt, Maria Manuela Ferreira Pereira da Silva Martins, Letícia de Lima Trindade, Olga Maria Pimenta Lopes Ribeiro, João Miguel Almeida Ventura-Silva, Vanessa Silva Corralo

**Affiliations:** 1 Postgraduate Program in Health Sciences, Regional Community University of Chapecó, Chapecó, Brazil; 2 Abel Salazar Institute of Biomedical Sciences, University of Porto, Porto, Portugal; 3 State University of Santa Catarina, Chapecó, Brazil; 4 RISE-Health, Nursing School, University of Porto, Porto, Portugal; 5 Higher School of Health of the Northern Region of the Portuguese Red Cross, Oliveira de Azeméis, Portugal

**Keywords:** fatigue, healthcare workforce, nursing, professional performance, working conditions

## Abstract

**Introduction:**

Occupational fatigue among nursing professionals is a multifactorial and complex phenomenon, with significant repercussions for both health and professional performance. In light of this scenario, Work Capacity (WC) emerges as an important indicator of job sustainability, reflecting the balance between individual resources and occupational demands.

**Objective:**

To analyze the relationship between fatigue levels and work capacity and their associations with sociodemographic, occupational, and health characteristics in nursing professionals from Brazil and Portugal. Methods: This cross-sectional, analytical, and comparative study involved 579 nursing professionals, 370 from Brazil and 209 from Portugal. Sociodemographic, work-related, and health variables were collected, in addition to the application of the Fatigue Assessment Scale (FAS) and the Work Ability Index (WAI). Descriptive and inferential statistical analysis was performed.

**Results:**

The prevalence of fatigue was high in both countries, reaching approximately 70% of participants, with a higher proportion of severe fatigue in Brazil (*p* = 0.030). In the subdomains, Brazilians presented greater mental fatigue, and Portuguese presented greater physical fatigue (*p* < 0.05). Work capacity was lower in Portugal (*p* < 0.001). A significant inverse correlation was observed between fatigue and WC in both countries (*p* < 0.001).

**Conclusion:**

Fatigue is high in Nursing and shows a consistent inverse relationship with work capacity in different national contexts. There is a clear need for organizational and occupational health interventions aimed at reducing fatigue, improving sleep, mitigating occupational stressors, and promoting job sustainability in nursing.

## Introduction

Occupational fatigue has been recognized as a multifactorial and complex phenomenon associated with high work demands and the organizational conditions in which work is performed (1–3). For healthcare professionals, who bear responsibility for the lives and wellbeing of others, fatigue is particularly relevant, as it extends beyond individual experience and impacts professional performance and, therefore, the functioning of healthcare systems (4–6).

In the context of Nursing, fatigue results from the dynamic interaction between work-related and individual factors. The high physical, cognitive, and emotional demands of the job, generated by the need for constant vigilance, continuous exposure to critical events, decision-making under pressure, simultaneous management of multiple demands, and constant exposure to suffering, pain, and death, combine with personal characteristics such as the worker’s health status, age, sleep quality, and individual coping mechanisms, triggering progressive exhaustion (1–3, 5). In contexts marked by resource shortages, long working hours, a high patient-to-professional ratio, and shift work, the demands of nursing work tend to produce a significant human cost associated with care, often manifested as fatigue (1). In addition, the high proportion of female professionals in the workforce should be considered, as evidence indicates that fatigue levels are significantly higher among women compared to men (7).

In practical terms, fatigue can compromise sustained attention, reaction time, decision-making, and psychomotor performance, with implications for patient safety and worker health (3, 5, 8). Factors such as workload, stress, and sleep deprivation are among the main determinants of adverse outcomes related to nursing performance, including medication errors and perceptions of poorer quality of care (1, 3, 8). Shift work, which is common in nursing, negatively affects not only performance but also workers’ wellbeing, as it increases fatigue levels. There is consistent evidence that professionals’ wellbeing plays an important role in the quality and safety of care (9, 10). Workloads exceeding 40 h per week and the performance of overtime have been associated with decreased work performance and poorer patient safety outcomes across occupational sectors (10).

From this perspective, work capacity (WC) constitutes an indicator for monitoring the cumulative effects of fatigue, allowing for organizational interventions in the Nursing work context (2, 11). Defined as the balance between a worker’s resources—including health, functionality, and mental resources—and the demands of the job, work capacity reflects the current level of performance and, above all, the potential for continued productive employment (12). Considering the global shortage of nursing professionals and the context of an aging workforce (4), understanding the relationship between occupational fatigue and WC is a guiding framework for policies on management, retention, and promotion of the health of the category.

Despite advances in the scientific literature on this topic, important limitations remain regarding the integrated analysis of occupational fatigue and work ability across different sociolabor contexts. In this sense, Brazil and Portugal constitute particularly relevant scenarios for analysis, since they present health systems, nursing workforce, and work models with certain specificities. In both countries, health services are organized through public systems with universal coverage, which are responsible for employing the largest share of the nursing workforce (13). However, in Brazil, the services of the Unified Health System (*Sistema Único de Saúde*, SUS) are completely free, financed by taxes, and encompass primary, secondary, and tertiary care in an integrated manner within Health Care Networks (*Redes de Atenção à Saúde*, RAS) (14, 15). From a slightly different perspective, Portugal has the National Health Service (*Serviço Nacional de Saúde*, SNS), which tends to be free of charge. The SNS is structured around primary, secondary, tertiary, and integrated continuing care services, based on Local Health Units (*Unidades Locais de Saúde*, ULS), and is financed by taxes and the collection of moderate fees for some types of consultations and emergency care (13).

The composition of nursing professionals within each country’s workforce presents even more significant differences, reflected in distinct work models (16, 17). In Brazil, the workforce encompasses professionals with varying levels of training (nursing assistants, nursing technicians, and nurses), each with specific responsibilities. Based on this, care follows a model of divided tasks and responsibilities in relation to the patient, adhering to the logic of teamwork (16). In the Portuguese context, nursing is structured exclusively by professionals with higher education degrees, with entry and career progression regulated through competitive examinations. The professional structure is organized into hierarchical categories according to experience and specialization, comprising general care nurses, specialist nurses, and nurse managers. From this perspective, the adopted work model is more individualized, in which the same professional undertakes the planning, execution, and assessment of care (17).

The differences between the contexts studied reveal structural issues that demonstrably affect different levels of healthcare delivery: professionals with lower levels of training may represent reduced labor costs in the face of budget constraints; however, they are associated with increased rates of preventable deaths and other adverse patient outcomes (18). Although this may be considered a strategy to address the shortage of nurses, less qualified professionals have been shown to have a higher risk of burnout and turnover compared to nurses (18).

In this context, analyzing the relationship between fatigue and work ability across different sociolabor contexts emerges as a strategy to deepen the understanding of the mechanisms that compromise wellbeing and performance in nursing. The comparison between Brazil and Portugal, considering their organizational and structural specificities, enables the identification of patterns and variations in the phenomenon, contributing to a more integrated understanding of its determinants. In this sense, the present study aims to analyze the relationship between fatigue levels and work capacity and their associations with sociodemographic, work-related, and health characteristics in nursing professionals from Brazil and Portugal.

## Materials and methods

This is a cross-sectional, analytical, and comparative study conducted with nursing professionals working in Brazil and Portugal. To ensure methodological accuracy, quality, and transparency of scientific writing, the Strengthening the Reporting of Observational Studies in Epidemiology (STROBE®) tool was used (19). The sampling was non-probabilistic, based on convenience, a strategy adopted due to the operational infeasibility of accessing comparable probabilistic sampling frames in Brazil and Portugal.

The study included 579 nursing professionals, of which 370 were Brazilian, including nursing assistants, nursing technicians, and nurses, and 209 were Portuguese, encompassing general care nurses, specialists, and managers. The sample composition took into account the nursing organization in each country, since in Brazil, the team consists of mid-level, technical, and higher-level professionals, while in Portugal, all nursing professionals are higher-level, distributed across the three described categories.

The inclusion criteria adopted were the practice of the profession in any type of health service in the respective countries. The exclusion criteria considered were professionals working outside the health sector and those who submitted incomplete questionnaires, making it impossible to analyze the results according to the proposed methodology. All participants who fully completed the fatigue questionnaire were considered for specific fatigue analysis, and the same procedure was adopted for the analysis of Work Capacity (WC). To assess the association between fatigue and WC, participants who fully completed both questionnaires were included.

Data collection was carried out with institutional support from the Federal Nursing Council (*Conselho Federal de Enfermagem*, Cofen) and the Regional Nursing Councils (*Conselhos Regionais de Enfermagem*, Coren) in Brazil, and from the São João Local Health Unit (ULS São João), linked to the National Health Service (SNS) in Portugal. The study was disseminated through institutional channels, social media, and scientific events focused on nursing in both countries. Participation was strongly encouraged among older professionals with longer experience, in order to highlight the potential impacts of aging on the workforce. Data collection took place between May 2024 and February 2025.

The data were collected through an online questionnaire made available on the Survey Monkey platform. To minimize bias associated with online data collection, the following strategies were adopted: the estimated completion time and inclusion criteria were described on the initial page of the platform; multiple recruitment channels were used (email, social media, institutional websites, professional groups, and scientific events); the instrument was designed using clear, objective, and easily understandable language; and the survey format allowed access across different devices (mobile phones, computers, and tablets). Controlling for duplicate responses and verifying inconsistent information in the online questionnaire were procedures adopted and conducted independently by two researchers. The instrument comprised a profile questionnaire with closed and mixed questions, addressing sociodemographic, work-related, and health aspects. Two instruments validated in both countries were applied: the Work Ability Index (WAI) (12, 20–22) and the Fatigue Assessment Scale (FAS) (23–25).

The WAI consists of questions that consider the worker’s resources and health, based on the professional’s own perception. The questionnaire is structured, with a total score ranging from 7 to 49 points, and has seven dimensions, which include: (1) Current work capacity; (2) Work capacity, considering physical and mental demands; (3) Self-reported and diagnosed illnesses; (4) Estimated productivity related to the presence of illnesses or injuries; (5) Number of absences from work due to illness; (6) Projected work capacity; and (7) Mental resources. Based on the score (7–49), the professional is classified according to their work capacity: low (≥27), moderate (28–36), good (37–43), and excellent (44–49) (12).

The version of the WAI used in this study was validated for the Portuguese language and demonstrated internal consistency, with a Cronbach’s alpha coefficient of 0.72 and, in the most recent reassessment, 0.787 (21, 22).

The FAS consists of 10 questions that assess the presence of chronic fatigue, based on statements that must be categorized according to their frequency of occurrence. Each statement is assessed on a five-point Likert scale, ranging from 1 (never) to 5 (always), with a total score ranging from 10 to 50 points. The result is considered from two subscales: mental fatigue and physical fatigue. According to the total score presented, the level of fatigue is considered: normal (<22), mild to moderate fatigue (22–34), and severe fatigue (≥35) (23).

The psychometric properties of the original FAS demonstrated strong internal consistency, with Cronbach’s alpha coefficients ranging from 0.87 to 0.90 across multiple samples (23, 26–28). For this study, culturally adapted and validated versions of the scale were used for Brazil (24) and Portugal (25). The Brazilian version showed internal consistency with Cronbach’s alpha coefficients of 0.80 and 0.85 across the two groups analyzed, while the Portuguese version presented a Cronbach’s alpha of 0.87 (24, 25).

Data analysis was performed using descriptive, comparative, and inferential statistics in the statistical Software Le Sphinx Plus2 (version 4.5) and IBM® SPSS® (Version 26.0). Results are described using absolute (n) and relative (%) frequencies, arithmetic means, and their respective standard deviations, minimum and maximum values, median, percentile ranks (P25 and P75), and interquartile range (IQR), according to the nature of the variables. The Kolmogorov–Smirnov test was used to test the normality of the scores. The Mann–Whitney test was used to compare scores between countries. Pearson’s chi-square (*χ*^2^) test was used to test for associations between variables. The association between fatigue and work capacity was analyzed using Spearman’s correlation coefficient (*ρ*), due to the non-normality of the variables, and the results were presented in a scatter plot with a trend line. For all analyses, a result was considered significant when the value was equal to or greater than 95% (1-*p* ≥ 95.0%), that is, *p* ≤ 0.05.

The study was approved by the Ethics Committee of Brazil (opinion no. 6885885/2024) and Portugal (opinion no. 254/2024). All participants voluntarily consented to participate in the study by signing the Free and Informed Consent Form. The data were treated anonymously and confidentially, in accordance with the ethical principles in force in both countries.

## Results

The results regarding fatigue levels and work capacity are detailed in Table 1.

**Table 1:** Fatigue level (*n* = 579) and work capacity (*n* = 534) of nursing professionals from Brazil and Portugal.

	Brazil		Portugal		*p*
	*n*	%	*n*	%	
Fatigue level					
Severe	59	15.9	18	8.6	0.030*
Mild to moderate	193	52.2	126	60.3	
Normal	118	31.9	65	31.1	
Total	370	63.9	209	36.1	
Work capacity					
Low	35	10.4	40	20.1	<0.001*
Moderate	142	42.4	102	51.3	
Good	109	32.5	50	25.1	
Excellent	49	14.6	7	3.5	
Total	335	62.7	199	37.3	

*Chi-square test (*χ*^2^).

The prevalence of fatigue among nursing professionals reached approximately 70%, both in Brazil and Portugal. Severe fatigue was positively associated with Brazilians and negatively associated with Portuguese. Regarding the assessment of WC, slightly more than half of the Brazilians presented negative outcomes (low or moderate WC), while among the Portuguese, this proportion exceeded 70% of nursing professionals.

In the analysis of the Fatigue subgroups (Table 2), greater mental fatigue was identified among Brazilians (*p* = 0.003) and greater physical fatigue among Portuguese (*p* = 0.021).

**Table 2:** Fatigue (mental, physical, and total) and work capacity (WAI) in nursing professionals from Brazil and Portugal.

Country		*N*	$\bar{x}$	σ	$M_{e}$	Minimum	Maximum	Percentiles		
								P25:	P75:	IQR
Brazil	Mental Fatigue^(a)^	370	12.1	4.4	11.0	5.0	25.0	9.0	15.0	6.0
	Physical Fatigue^(b)^	370	13.8	4.3	14.0	5.0	24.0	11.0	17.0	6.0
	Fatigue^(c)^	370	25.9	8.2	24.0	10.0	48.0	20.0	31.0	11.0
	WAI^(d)^	335	36.0	6.8	36.0	15.0	49.0	31.0	42.0	11.0
Portugal	Mental Fatigue^(a)^	209	10.8	3.3	10.0	5.0	21.0	9.0	13.0	4.0
	Physical Fatigue^(b)^	209	14.6	3.9	15.0	5.0	25.0	11.5	18.0	6.5
	Fatigue^(c)^	209	25.4	6.6	25.0	12.0	46.0	20.0	30.0	10.0
	WAI^(d)^	199	32.7	6.2	33.0	12.0	45.0	29.0	37.0	8.0

$\bar{x}$ = arithmetic mean; σ = standard deviation; *M_e_* = median; IQR = interquartile range. (a) *p* = 0.003; (b) *p* = 0.021; (c) *p* = 0.907; (d) *p* < 0.001. Mann–Whitney test.

Table 3 shows which variables were associated with Fatigue in each country.

**Table 3:** Association between fatigue and sociodemographic, work-related, and health variables in Brazil (*n* = 370) and Portugal (*n* = 209).

Variable		Brazil			Portugal		
Sociodemographic		Relative frequency (%)	Chi-square (*χ*^2^)	p*	Relative frequency (%)	Chi-square (*χ*^2^)	p*
Gender	Female	84.6	5.75	0.056	80.4	15.1	**0.001**
	Male	15.4			19.6		
Age group	20–34	19.5	17.70	**0.001**	10.5	1.15	0.887
	35–49	47.8			51.7		
	50 or older	32.7			37.8		
Marital status	Married/in a stable union	60.0	6.41	0.379	71.3	9.75	0.136
	Single	24.6			18.7		
	Divorced	13.0			9.1		
	Widowed or Other	2.4			0.9		
Children	None	27.6	7.83	0.251	26.3	9.23	0.161
	1 child	27.8			21.5		
	2 children	29.5			43.5		
	3 or more children	15.1			8.7		
Housing arrangement	Living with Family	83.2	6.04	0.196	85.6	2.23	0.693
	Living alone	15.4			11.5		
	Other	1.4			2.9		
Labor							
Schooling level	High school/technical education	27.6	10.32	0.243	0.0	3.95	0.684
	Higher education	17.3			22.0		
	Specialization	40.8			39.3		
	Master’s degree	11.9			37.3		
	Doctoral degree	2.4			1.4		
Professional category**	Nursing assistant	1.9	2.98	0.562	-	–	–
	Nursing technician	40.1			-		
	Nurse	58.0			-		
	General nurse	–	–	–	29.2	8.58	0.073
	Specialist nurse	–			58.8		
	Nurse manager	–			12.0		
Work setting	Hospital setting	50.5	10.84	0.094	73.2	11.06	**0.026**
	Primary Health Care	42.2			26.3		
	Other***	5.4			0.5		
	Two settings	1.9			0.0		
Function	Clinical	74.3	4.00	0.407	72.7	7.12	0.130
	Managerial	19.2			21.5		
	Clinical and managerial	6.5			5.8		
Length of experience	Less than 10 years	10.5	13.63	**0.009**	6.2	2.49	0.656
	10–20 years	50.5			36.4		
	More than 20 years	38.6			57.4		
Number of employment relationships	One job	74.6	3.98	0.408	71.8	2.08	0.721
	Two Jobs	24.0			25.8		
	Three or more jobs	1.4			2.4		
Type of contract	Public servant	45.9	6.67	0.756	33.6	5.84	0.655
	Permanent employee (formal contract)	33.9			56.8		
	Temporary employee (formal contract)	4.5			2.2		
	Informal employee (no formal contract)	7.5			0.0		
	Self-employed	5.5			5.7		
	Other	2.7			1.7		
Workload	Less than 40 h per week	26.0	10.97	**0.027**	57.9	2.79	0.593
	40–59 h per week	58.0			39.7		
	60 h or mores	16.0			2.4		
Earnings****	Less than 2 minimum wage	11.9	3.28	0.772	36.8	8.14	0.087
	2–4 minimum wage	44.6			60.8		
	4–6 minimum wage	28.6			2.4		
	More than 6 minimum wage	14.9			0.0		
Health							
Chronic disease	No	62.2	4.94	0.084	58.0	0.83	0.659
	Yes	37.8			42.0		
Medication use	No	44.3	10.53	**0.005**	49.8	3.37	0.186
	Yes	55.7			50.2		
Health treatment	No	48.0	18.81	**0.001**	56.0	5.01	0.286
	Yes, pharmacological treatment	39.0			32.1		
	Yes, other treatment	13.0			11.9		
Physical activity	No	27.6	9.31	**0.009**	25.4	0.12	0.944
	Yes	72.4			74.6		
Self-assessment of diet	Good or excellent	49.8	17.62	**0.001**	45.9	15.13	**0.004**
	Fair	38.0			47.4		
	Poor or very poor	12.2			6.7		
Sleep quality	Good or excellent	40.6	51.54	**<0.001**	23.9	19.86	**0.001**
	Fair	37.7			41.6		
	Poor or very poor	21.7			34.5		
Stress risk	Low or very low	5.4	62.99	**<0.001**	1.0	20.90	**0.001**
	Moderate	35.4			22.6		
	High or very high	59.2			76.4		
Self-perception of physical health	Good or excellent	52.2	106.32	**<0.001**	44.0	30.67	**<0.001**
	Fair	35.1			44.0		
	Poor or very poor	12.7			12.0		
Self-perception of mental health	Good or excellent	46.3	154.04	**<0.001**	42.1	45.51	**<0.001**
	Fair	39.9			42.6		
	Poor or very poor	13.8			15.3		

*Chi-square test (*χ*^2^). *p*-values < 0.05 indicate a statistically significant association.

**The professional category is presented according to the workforce organization of each country.

***The category “Other” includes laboratories, clinics, home care services, specialized care, occupational health, health management, long-term care institutions for older adults, rehabilitation, and private health services.

****The Brazilian minimum wage ranged from R$1.412,00 (2024) to R$1.518,00 (2025) during the data collection period. The Portuguese minimum wage ranged from €820 to €870 over the same period.

Bold values indicate statistically significant (*p* < 0.05).

Among sociodemographic variables, fatigue was associated with gender among the Portuguese, with better results among men. Among Brazilians, fatigue was associated with age: professionals aged 50 or older were positively associated with normal levels and negatively associated with severe fatigue. Furthermore, the 20–34 age group was negatively associated with normal levels of fatigue.

Regarding work variables, Portugal showed an association between fatigue and the field of work, reporting that professionals in other fields (other than primary care and/or hospitals) had a positive association with severe fatigue. In Brazil, the length of experience and workload were associated with fatigue. Professionals with more than 20 years of experience were positively associated with normal levels, while those who had been working for less than 10 years were positively associated with severe fatigue. Regarding workload, there was a positive association between the group working less than 40 h per week and normal levels of fatigue. The group working more than 60 h per week was positively associated with severe fatigue.

Health variables showed a close relationship with fatigue levels among nursing professionals. In Brazil, medication use was negatively associated with normal fatigue levels. Among professionals who did not use medication, a positive association was identified with normal levels and a negative association with severe fatigue. Similarly, Brazilians who do not undergo any type of health treatment showed a positive association with normal levels.

Regarding the assessment of physical activity practice by nursing professionals and its relationship with fatigue levels, a positive association was observed among Brazilians between sedentary individuals and severe fatigue, while a negative association was found between sedentary individuals and normal fatigue levels.

The variables self-assessment of diet, sleep quality, stress risk, physical and mental health were associated with fatigue in both countries. The worst assessments of these variables were positively associated with severe fatigue in Brazil. In Portugal, the best assessments of these variables were positively associated with normal fatigue levels.

Table 4 shows which variables were associated with Work Capacity in each country.

**Table 4:** Association between work capacity and sociodemographic, work-related, and health variables in Brazil (*n* = 335) and Portugal (*n* = 199).

Variable		Brazil			Portugal		
Sociodemographic		Relative frequency (%)	Chi-square (*χ*^2^)	p*	Relative frequency (%)	Chi-square (*χ*^2^)	p*
Gender	Female	85.4	6.14	0.105	80.9	13.73	**0.003**
	Male	14.6			19.1		
Age group	20–34	17.6	5.79	0.448	9.5	9.84	0.132
	35–49	49.6			53.8		
	50 or older	32.8			36.7		
Marital status	Married/in a stable union	60.6	9.33	0.407	71.4	7.22	0.614
	Single	24.5			19.1		
	Divorced	12.2			8.5		
	Widowed or Other	2.7			1.0		
Children	None	26.6	10.17	0.337	26.6	3.98	0.913
	1 child	28.4			21.6		
	2 children	29.8			43.8		
	3 or more children	15.2			8.0		
Housing arrangement	Living with Family	83.3	5.28	0.508	85.4	7.39	0.286
	Living alone	14.9			12.1		
	Other	1.8			2.5		
Labor							
Schooling level	High school/technical education	27.2	13.63	0.325	0.0	5.48	0.791
	Higher education	16.7			2.6		
	Specialization	41.2			40.2		
	Master’s degree	12.5			36.7		
	Doctoral degree	2.4			1.5		
Professional category**	Nursing assistant	2.7	11.80	0.066	–	–	–
	Nursing technician	38.6			–		
	Nurse	58.7			–		
	General nurse	–	–	–	28.6	7.91	0.245
	Specialist nurse	–			59.8		
	Nurse manager	–			11.6		
Work setting	Hospital setting	51.0	12.53	0.185	73.4	5.52	0.479
	Primary Health Care	41.5			26.1		
	Other***	5.7			0.5		
	Two settings	1.8			0.0		
Function	Clinical	74.6	9.03	0.172	72.4	3.00	0.809
	Managerial	19.1			21.1		
	Clinical and managerial	6.3			6.5		
Length of experience	Less than 10 years	10.8	6.10	0.412	5.5	10.62	0.101
	10–20 years	50.7			36.7		
	More than 20 years	38.5			57.8		
Number of employment relationships	One job	74.0	10.47	0.106	70.9	2.85	0.828
	Two Jobs	24.5			26.6		
	Three or more jobs	1.5			2.5		
Type of contract	Public servant	45.9	11.23	0.736	32.9	14.31	0.282
	Permanent employee (formal contract)	34.6			57.1		
	Temporary employee (formal contract)	3.8			2.3		
	Informal employee (no formal contract)	6.9			0.0		
	Self-employed	6.1			5.9		
	Other	2.7			1.8		
Workload	Less than 40 h per week	24.8	6.57	0.363	56.8	3.04	0.804
	40–59 h per week	59.6			40.7		
	60 h or mores	15.6			2.5		
Earnings****	Less than 2 minimum wage	13.1	10.80	0.289	35.7	7.36	0.289
	2–4 minimum wage	41.5			61.8		
	4–6 minimum wage	29.6			2.5		
	More than 6 minimum wage	15.8			0.0		
Health							
Chronic disease	No	61.2	27.06	**<0.001**	56.8	21.70	**0.001**
	Yes	38.8			43.2		
Medication use	No	42.1	49.39	**<0.001**	48.2	19.79	**0.001**
	Yes	57.9			51.8		
Health treatment	No	45.5	78.71	**<0.001**	54.8	25.84	**0.001**
	Yes, pharmacological treatment	40.1			32.7		
	Yes, other treatment	14.4			12.6		
Physical activity	No	26.9	13.83	**0.003**	26.1	3.50	0.321
	Yes	73.1			73.9		
Self-assessment of diet	Good or excellent	49.1	17.98	**0.006**	45.2	6.76	0.344
	Fair	37.4			47.8		
	Poor or very poor	13.5			7.0		
Sleep quality	Good or excellent	38.3	34.90	**<0.001**	24.1	31.83	**<0.001**
	Fair	38.6			42.7		
	Poor or very poor	23.1			33.2		
Stress risk	Low or very low	4.8	32.84	**<0.001**	1.0	25.06	**0.001**
	Moderate	33.4			20.7		
	High or very high	61.8			78.3		
Self-perception of physical health	Good or excellent	50.4	91.90	**<0.001**	42.7	58.96	**<0.001**
	Fair	36.1			45.2		
	Poor or very poor	13.4			12.1		
Self-perception of mental health	Good or excellent	44.9	100.35	**<0.001**	41.2	35.39	**<0.001**
	Fair	41.0			42.7		
	Poor or very poor	14.1			16.1		

*Chi-square test (*χ*^2^). *p*-values < 0.05 indicate a statistically significant association.

**The professional category is presented according to the workforce organization of each country.

***The category “Other” includes laboratories, clinics, home care services, specialized care, occupational health, health management, long-term care institutions for older adults, rehabilitation, and private health services.

****The Brazilian minimum wage ranged from R$1.412,00 (2024) to R$1.518,00 (2025) during the data collection period. The Portuguese minimum wage ranged from €820 to €870 over the same period.

Bold values indicate statistically significant (*p* < 0.05).

The gender variable among Portuguese professionals drew attention because it was associated with both fatigue and WAI: being male was positively associated with normal levels of fatigue and excellent work capacity. Using gender as a variable, associations were observed that may be linked to better WC and fatigue results among Portuguese men: higher monthly earnings (*p* = 0.002), better mental health assessment (*p* = 0.001), better sleep quality (*p* = 0.003), and lower risk of stress (*p* = 0.044), however, with a higher weekly workload than women (*p* = 0.008).

Among Brazilians, there was no significant association between any sociodemographic variable and WC.

Regarding work-related variables, no associations were found with the WC of professionals from any of the countries analyzed.

Health variables showed a significant relationship with WC. In Brazil, all variables were associated with WC. In Portugal, only physical activity and self-assessment of diet were not associated with WC outcomes.

Low WC was associated with worse assessments of physical and mental health in both countries. Good WC was positively associated with better assessments of sleep and stress risk, both among Brazilians and Portuguese. In Brazil, excellent WC was positively associated with the absence of chronic disease, good or excellent diet, physical activity, non-use of medications, and non-use of any type of health treatment. In Portugal, low WC was positively associated with the presence of chronic disease, use of medications, and use of any type of health treatment.

**Figure 1:**
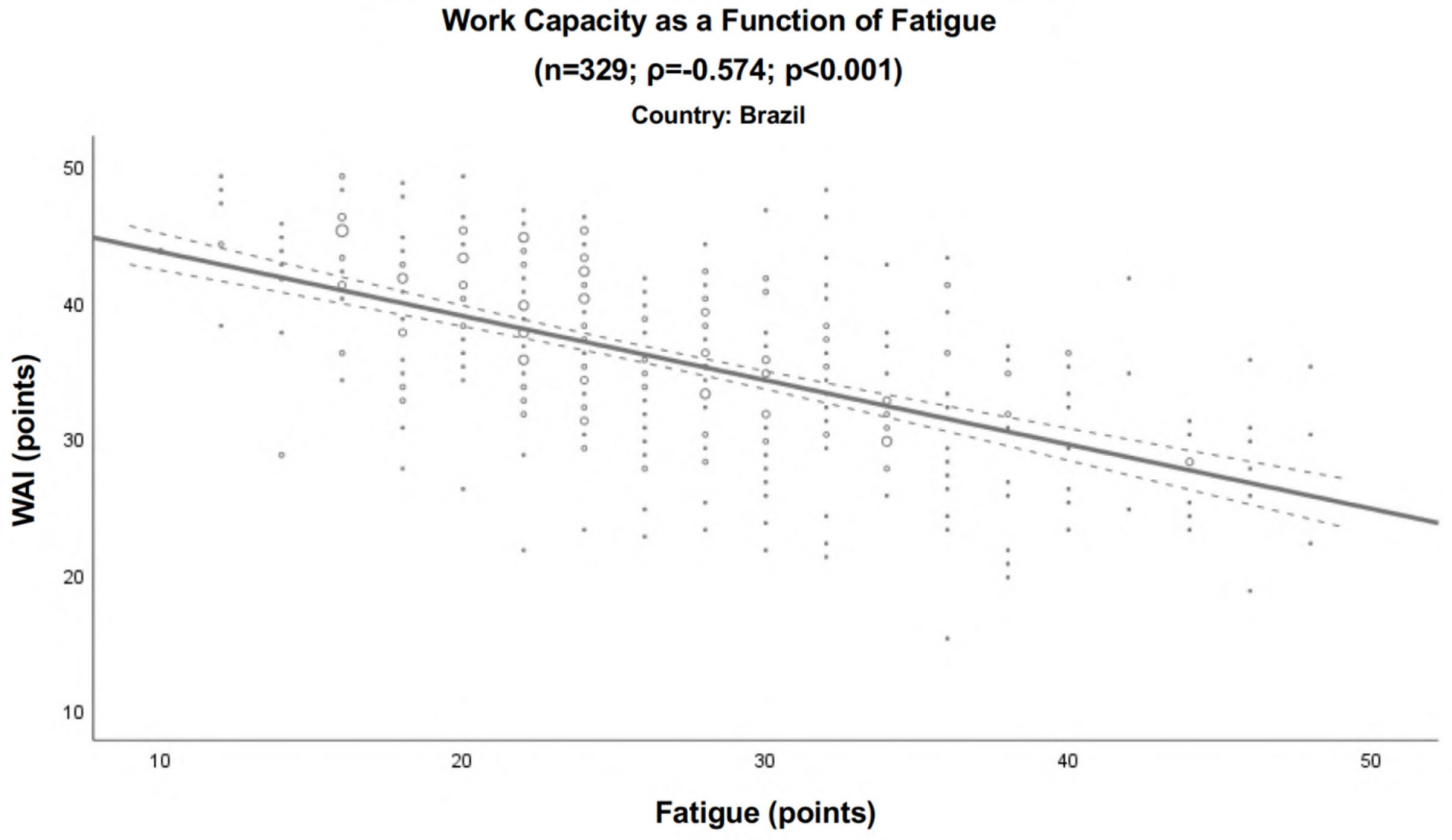
Relationship between fatigue and work capacity of nursing professionals in Brazil (*n* = 329).

In both Brazil and Portugal, an inversely significant association was observed between Fatigue and Work Capacity (*p* < 0.001). In Brazil (Figure 1), professionals with “excellent” capacity are associated with “normal” fatigue levels, while those with “moderate” and “good” capacity are associated with “mild to moderate” fatigue, and professionals with “low” capacity are associated with “severe” fatigue.

**Figure 2:**
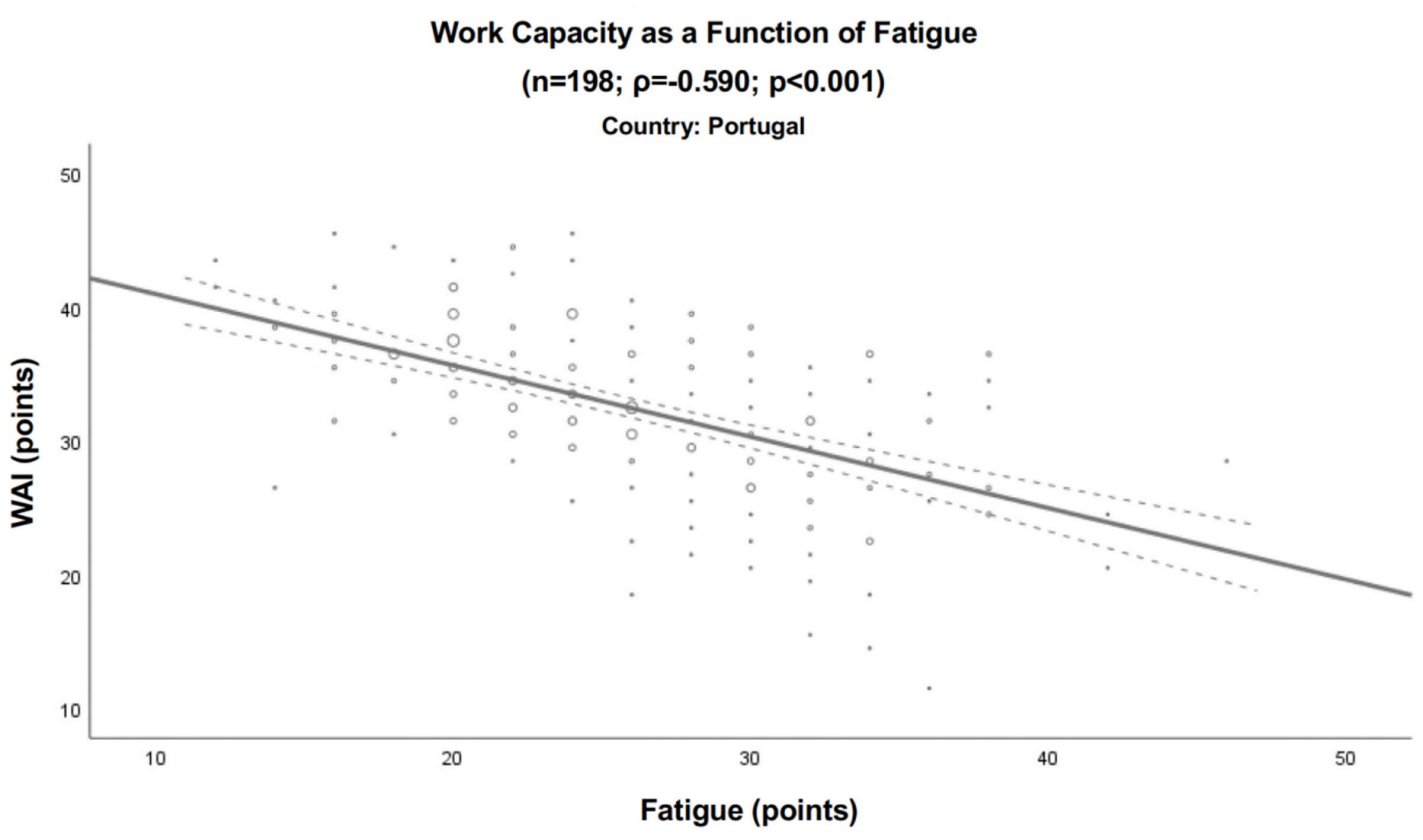
Relationship between fatigue and work capacity of nursing professionals in Portugal (*n* = 198).

Following the same trend, in Portugal (Figure 2), professionals with “good” and “excellent” capacity are associated with “normal” fatigue, those with “low” and “moderate” capacity are associated with “mild to moderate” fatigue, and professionals with “low” work capacity are associated with “severe” fatigue.

## Discussion

The results of this study highlight a critical phenomenon that has been little explored comparatively. By demonstrating a consistent inverse association between fatigue and work capacity, the results indicate that fatigue should not be understood as an isolated factor, but as an indicator of impaired work capacity. Analyzing this phenomenon among nursing professionals working within different work organization models, areas of practice, and national contexts, such as Brazil and Portugal, contributes to a broader understanding of the topic and reinforces the relevance of the study in an international context.

The high prevalence of fatigue identified in this study confirms the magnitude of the problem and reinforces the need to recognize it as an important risk factor for health and professional performance in Nursing in both countries studied. Although the frequency of fatigue was similar between the two countries, the higher prevalence of severe fatigue among Brazilian professionals points to a worrying scenario in this context, suggesting greater occupational vulnerability (2).

In Greece, a study with 404 nurses identified lower scores, with 60.4% reporting fatigue. Using the same assessment instrument, the authors also observed a lower average score (24.08), reinforcing the hypothesis that contextual, organizational, and work-related factors exert a significant influence on the intensity of fatigue among nursing professionals (29).

The literature indicates that fatigue tends to intensify with advancing age, due to the interaction between aging and occupational exhaustion (30). However, a study conducted in Poland with 424 palliative care nurses, with a mean age of 50.65 years, identified a mean score of 20.78 on the Fatigue Assessment Scale, a value substantially lower than that observed in the present study (31). Although there is evidence of increased fatigue with age, this finding suggests that the phenomenon cannot be explained solely by age, indicating the existence of other factors that are intertwined with the results of fatigue (31).

The comparison between the countries revealed distinct patterns of fatigue: mental fatigue predominated among Brazilians, while physical fatigue stood out among the Portuguese. Authors state that mental fatigue is associated with lower levels of resilience, while physical fatigue is more directly related to working conditions (32). In light of these findings, the higher mental fatigue among Brazilians may be associated with the younger age profile of the sample, considering that resilience is usually strongly linked to older and more experienced professionals (33, 34).

On the other hand, the greater physical fatigue identified among Portuguese professionals seems to align with two central aspects. The first refers to the significant representation of nurses working in the hospital setting, an environment known to be associated with a higher physical workload compared to other practice settings (35). The second relates to functional aging itself, since the physical demands of nursing practice become progressively more exhausting in older age groups (36).

Another important consideration involves the care setting of Brazilian and Portuguese nursing professionals, as there is evidence that the structure of the practice environment and the outcome of nursing care received better assessments in the Brazilian context, while the process involving nursing care was better assessed in the Portuguese setting (37). Issues such as work organization, job security, and regulation of working hours differ between countries and can contribute to understanding the outcomes of fatigue.

The main model of work organization in Portugal is more oriented toward the planning, execution, and assessment of care in an individualized manner by nursing professionals (17). This approach provides a broader view of the patient’s care trajectory, promoting greater autonomy and accountability. In Brazil, on the other hand, the model unfolds through the distribution of tasks and collaboration among team members. The perspective of teamwork favors operational efficiency; however, effective coordination is necessary (16).

Furthermore, labor laws regulating Nursing work stipulate significantly different work schedules between the two countries: 35 h per week for Portuguese workers and 44 h for Brazilian workers (38, 39). The evidence of a high proportion of precarious and multiple employment relationships among Brazilian nursing professionals paints an even more negative picture, given the significantly longer working hours compared to Portuguese nurses (40, 41). These distinctions contribute to understanding the different patterns of fatigue (mental and physical) presented by professionals from the two countries.

Regarding general fatigue, an association with age was found only among Brazilians, with a higher prevalence of fatigue among younger professionals, corroborating other studies (42, 43). In contrast, authors have identified a positive association between age and fatigue, highlighting the heterogeneity of the results and the importance of the work context in interpreting these findings (31).

Regarding gender, only Portugal showed an association with fatigue, consistent with the study by Sikaras et al. (29), which showed significantly higher scores among women. Evidence from Egypt indicates that women have almost twice the risk of severe fatigue compared to men (43), which may reflect gender inequalities in the distribution of tasks, double shifts, and psychosocial burden.

Length of experience was negatively correlated with fatigue among Brazilians, corroborating previous studies (31, 43). Evidence suggests that fatigue can decrease with length of experience as a consequence of adaptation to work (42). Resilience, which is the ability to adapt positively to situations, is strongly associated with lower fatigue scores (32).

The findings of this study and others point to generational differences in the development of resilience (44) and its relationship with professional experience, the latter of which needs to be observed beyond age and years of experience, but rather considering the experiences provided and sought by the worker. Organizational investments aimed at strengthening resilience can also be defining factors in exposure to fatigue.

Still considering the work-related aspects, the Brazilian sample showed a positive relationship between working hours and fatigue levels. Clearly, the excessive number of hours worked has an impact on this aspect, especially if we consider the group of professionals who work more than 60 h a week, a practice that is not very common among the Portuguese. The International Labour Organization (ILO) emphatically states that excessive workload—in terms of work demands and/or working hours—combined with lack of rest or poor sleep quality are among the main causes of fatigue (45).

In line with these findings, sleep quality correlated with fatigue levels. Studies show that both physical and mental fatigue are strongly associated with sleep impairment (42), with sleep disorders being a recurring factor in the genesis of fatigue among nursing professionals in different contexts (46–48).

Stress, which is intertwined with long working hours, insufficient rest, and compromised sleep, has been shown to be directly associated with fatigue, corroborating findings from previous studies conducted with nursing professionals (31, 42). Fatigue, in turn, has proven to be a significant factor impacting the health of professionals. The worst levels of fatigue were associated with a more negative perception of their own health status, as also evidenced by Jang et al. (42). These results are aligned with the systematic review with meta-analysis carried out by Pi et al. (1), which identified associations between occupational fatigue, age, sleep quality, stress, workload, and self-perception of health. The authors highlight that work overload, related to understaffing, results in greater stress, poorer sleep quality, and, consequently, disruptive circadian rhythms. From this perspective, there are negative health repercussions, especially for younger and less experienced professionals, as well as among older professionals, who tend to experience limitations associated with aging (1).

Given this evidence, occupational fatigue takes center stage in the analysis of the repercussions of an aging nursing workforce, since its effects directly impact work capacity. Fatigue is strongly linked to decreased productivity, impaired cognitive function, and an increased chance of errors, impacting patient safety and the outcomes of care provided (42, 49). Considering the above, the analysis of the effects of fatigue on WC is a fundamental indicator of the sustainability of professional practice in nursing.

In this context, the assessment of WC revealed worrying results: 52.8% of Brazilians and 71.4% of Portuguese showed some level of impairment in their work capacity (low or moderate WC). Significant findings were also observed in a study conducted in Ethiopia, in which 59% of nurses presented low WC (50). In Brazil, a study identified a similar proportion of compromised WC (low or moderate) (51). In contrast, another Brazilian study, involving only nursing professionals working in public hospitals, did not identify cases of low WC, with the distribution concentrated between moderate WC (27.5%), good (51%), and excellent (21.5%) (52). The authors linked the findings to the participants’ long professional trajectories, which tend to provide coping strategies for better handling work demands (52).

The WAI averages observed in this study were lower than those reported in countries such as Italy and Tunisia (53, 54), approaching the values found in Egypt (50). Consistent with the findings from Portugal, one study found significantly worse WC scores among women compared to men (55). In fact, male nurses are 2.43 times more likely to have greater work capacity than female nurses (50).

The absence of a significant association between age and WC in this study corroborates national findings (52), although other authors have identified an inverse relationship between these variables (55). These discrepancies can be largely attributed to differences in work contexts, including the type of service, work organization, working conditions, and team size. Hospital professionals over 40 years of age, especially those in direct care activities, show a higher frequency of inadequate WC, reinforcing the mediating role of the work context (56).

Even more evidently than fatigue, WC showed a strong association with health variables. Better health assessments were directly related to higher WC scores, highlighting the centrality of health in work sustainability. Nurses with chronic illnesses are less likely to maintain elevated WC levels, as are those with poor sleep quality (50).

This study demonstrated a statistically significant inverse association between fatigue levels and work capacity in nursing professionals in both Brazil and Portugal, corroborating evidence linking occupational fatigue to reduced WC (11, 31, 57, 58). Organizational factors (workload, work demands, night shift, longer tenure in the same role and job category), individual factors (chronic diseases), and contextual factors emerge as common determinants of these outcomes, reinforcing the need for integrated approaches (11, 57, 58). These factors have also been associated with negative wellbeing outcomes and higher turnover in nursing. Evidence suggests that decent work is positively associated with nurses’ work capacity, being mediated by psychological wellbeing (59).

Clearly, the differences observed between Brazil and Portugal reflect the dynamic interaction of variables throughout the work cycle. In Brazil, the higher prevalence of multiple jobs and long working hours seems to contribute to greater fatigue and worse WC, while in Portugal the predominance of the hospital setting and the age profile may explain the greater physical fatigue. These findings point to the need for contextualized interventions tailored to the specificities of each setting. Occupational health policies in nursing should move beyond approaches focused solely on reducing workloads, incorporating continuous monitoring systems for wellbeing and fatigue, revising work schedules and shifts, ensuring adequate staffing levels, and implementing institutional recovery mechanisms, such as scheduled breaks and mental health support. Additionally, the results call for context-sensitive responses that consider the different configurations of health systems, including both workforce characteristics and models of care organization.

As a contribution, this study broadens the understanding of the factors associated with fatigue and work capacity in nursing professionals from two distinct contexts, highlighting the close relationship between these phenomena. The findings reinforce that the sustainability of nursing fundamentally depends on investment in human capital, especially in a scenario of progressive aging of the profession. By recognizing that there is no safe and quality care without healthy workers, this study reaffirms the importance of the nursing professional as a structuring axis of patient safety and quality of care.

A key strength of this study is its international comparative approach, combined with the use of validated instruments and the integrated analysis of sociodemographic, work-related, and health variables. Among the study limitations, the cross-sectional design precludes the establishment of causal relationships, limiting inferences to associations. In addition, convenience sampling may restrict the representativeness of participants and the generalizability of the findings. Online data collection may introduce selection bias, with greater participation from individuals more familiar with digital tools and/or more interested in the topic under investigation. Furthermore, information bias cannot be ruled out, as data were self-reported and therefore subject to subjective interpretation of the questions.

## Conclusion

The results show that fatigue is a frequent problem among nursing professionals in Brazil and Portugal, and that it is inversely and consistently associated with work capacity. Although the overall prevalence of fatigue was similar between the countries, Brazil presented a higher proportion of severe fatigue, while Portugal had the worst classifications of work capacity, suggesting that contextual characteristics (age profile, predominant sector of activity, and work organization) may influence specific patterns of the phenomenon.

From an applied perspective, the findings support the adoption of measures to manage fatigue risk and promote worker health in nursing, with a focus on: organizing workdays with rest breaks, carefully assessing the demands placed on night shifts, adjusting staffing levels, early identification of groups and sectors at greater risk, including reserve staff to cover absences and peaks in demand, adjusting duties for professionals with compromised WC, and strengthening institutional support throughout the work cycle.

Finally, by comparing two national contexts with distinct configurations of the nursing workforce, the study broadens the understanding of the determinants of occupational fatigue and work capacity, and reinforces the centrality of investment in working conditions and occupational health. The sustainability of the category is strongly linked to the health conditions of professionals, as the quality of care and patient safety are directly conditioned by the physical and mental conditions of those who provide care.

As recommendations for future research, there is a need for longitudinal studies to elucidate temporality and potential causal mechanisms underlying the relationship between fatigue and work ability. Additionally, studies incorporating broader organizational variables, such as institutional support, team climate, and safety culture, may contribute to a more comprehensive understanding of protective factors for wellbeing and the sustainability of the nursing workforce.

## Data Availability

The raw data supporting the conclusions of this article will be made available by the authors, without undue reservation.
